# Herbopathy-induced Cephalalgia: Remedy gone wrong

**Published:** 2019-07-06

**Authors:** Ashima Mittal, Alessandro Iliceto, Balaji Yegneswaran

**Affiliations:** Department of Internal Medicine, Saint Peter's University Hospital, New Brunswick, New Jersey, United States

**Keywords:** Cimicifuga, Headache, Herbal

The use of complementary and alternative medicine (CAM) has become widespread in many countries including the United States.^1^ Black cohosh, also known as Cimicifuga racemosa, is a botanical that has gained popularity over the past decades, and is currently prescribed by gynecologists for management of menopausal symptoms.^2^ Limited understanding of the therapeutic benefits, dosing guidelines, and adverse effects may present a risk for patients consuming the product. 

We report a 49-year-old woman with past medical history of pemphigus vulgaris presented to the Emergency Department (ED) with one-week history of diffuse headaches. Headache was described as 2/10 in intensity, diffuse, radiating to neck, and not relieved by ibuprofen. The prior week, the patient was seen by her primary physician, and was informed that hypertension was the likely cause of her headache. On the morning of presentation, the patient reported a coughing episode followed by headache that suddenly worsened to excruciating 10/10 intensity, described as if her head was splitting into two parts, worsened by movement, bending, not associated with lacrimation or rhinorrhea. The headache was partially relieved after taking hydrochlorothiazide (HCTZ). Home medications were black cohosh 540 mg daily that the patient had been taking since August 2018 for relief of menopausal symptoms. On physical examination, vital signs were significant for blood pressure of 150/70 mmHg and regular heart rate of 98 per minute. Visual inspection revealed an obese woman in distress with severe diffuse headache and intact neurological examination except for papilledema bilaterally observed on fundoscopy. Evaluation of inherited thrombophilia-anticardiolipin antibody, lupus anticoagulant, prothrombin G202108, protein C, protein S, and factor V Leiden was unremarkable. Magnetic resonance venography (MRV) of the head showed superior sagittal (Figure 1) and right transverse sinus thrombosis (Figure 2). The patient received a dose of low-molecular-weight heparin along with pain and nausea medications with gradual improvement of the headache. She was transferred to the intensive care unit (ICU) where heparin drip was started. 

**Figure 1:**
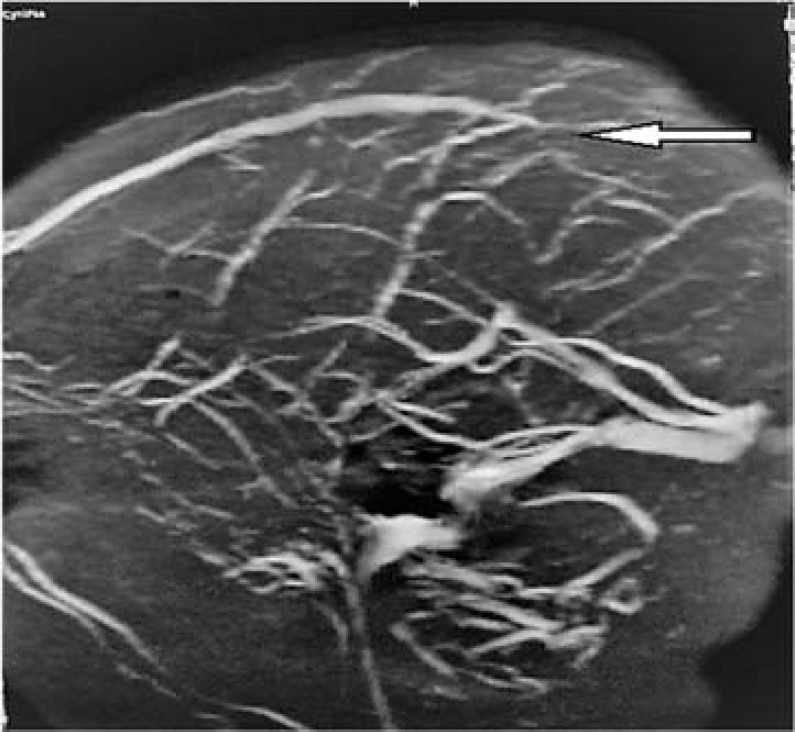
Superior sagittal sinus thrombosis (arrow) in magnetic resonance venography (MRV)

The patient was discharged in stable condition on oral Coumadin.

**Figure 2:**
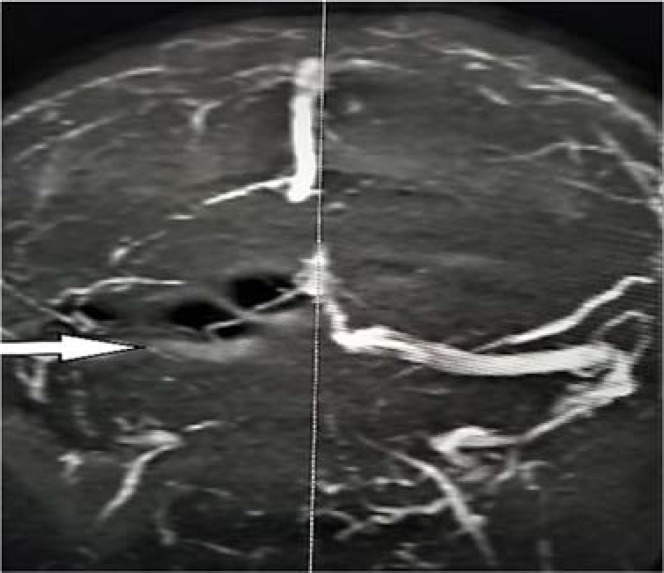
Right transverse sinus thrombosis (arrow) in magnetic resonance venography (MRV)

Black cohosh, a member of the buttercup family, originally found in North America, has been traditionally used by Chinese herbalists for management of various ailments. It is also known among different cultural groups as snakeroot, black bugbane, rattle weed, macrotys, and rheumatism weed.^2^

It has now become one of the most common herbal remedies recommended for mitigation of menopausal symptoms.^3^ Its root or rhizome is used for herbal preparation. The mechanism of action appears to be multifactorial with predominantly serotonergic partial agonist activity.^4^

A growing body of evidence suggests coagulative properties of the remedy leading to possible ischemic events.^5^ A systematic review evaluating the side effects of black cohosh mentions the most commonly observed adverse reactions of patients consuming the product included hepatotoxicity, herb-drug interaction, cardiovascular disease, and estrogenic effects particularly on the breast.^1^

Our patient presented with unexplained superior sagittal and right transverse sinus thrombosis after daily consumption of black cohosh. To the best of our knowledge, this is the first reported case available in the literature of cortical venous thrombosis as a side effect of black cohosh. The case should raise awareness of potential adverse effects of the herbal remedy, which should be considered when prescribing the product particularly in patients with underlying coagulopathies. Despite a recent increase in the use of herbal remedies, concerns regarding their potential adverse effects needs to be addressed. Randomized controlled clinical trials are needed to establish safety and efficacy of black cohosh definitively.

## Notes:

**How to cite this article: **Mittal A, Iliceto A, Yegneswaran B. Herbopathy-induced Cephalalgia: Remedy gone wrong. Iran J Neurol 2019; 18(3): 148-9.

## References

[1] Borrelli F, Ernst E (2008). Black cohosh (Cimicifuga racemosa): A systematic review of adverse events. Am J Obstet Gynecol.

[2] Gardner Z, McGuffin M (2013). American Herbal Products Association's botanical safety handbook.

[3] Geller SE, Shulman LP, van Breemen RB, Banuvar S, Zhou Y, Epstein G (2009). Safety and efficacy of black cohosh and red clover for the management of vasomotor symptoms: A randomized controlled trial. Menopause.

[4] Burdette JE, Liu J, Chen SN, Fabricant DS, Piersen CE, Barker EL (2003). Black cohosh acts as a mixed competitive ligand and partial agonist of the serotonin receptor. J Agric Food Chem.

[5] Zimmermann R, Witte A, Voll RE, Strobel J, Frieser M (2010). Coagulation activation and fluid retention associated with the use of black cohosh: a case study. Climacteric.

